# The Saturday Fund and the Hospitals

**Published:** 1913-02-22

**Authors:** G. A. Lewcock

**Affiliations:** 54 Solon Road, Brixton.


### The Saturday Fund and the Hospitals.

*To the Editor of* The Hospital.

Sir,—Before dealing with the replies to my letter of February 8, I should like to state that I am not representing the Hospital Saturday Fund in this matter, but acting entirely by myself.

Dr. A. C. E. Gray says I "have raised a point of very great importance to hospital committees and the cause of charity in general." I think it is time the question was raised, because our subscribers ought to be correctly informed of the connection of the Saturday Fund with the hospitals. The doctor refers to my question respecting the treatment of our subscribers. I must say that the working man interprets the letter to mean that he is entitled to treatment. At the same time my question must be taken in conjunction with the extracts I quote from the articles of association, and I am calling attention to the construction of these articles. I do not enter into the question of the adequacy of payment.

The Source of "Purchase."

Mr. Conrad W. Thies points out that hospital managers very fully appreciate the financial assistance received from the Saturday Fund and he also attributes to myself the word "purchase." If he will kindly refer to my letter he will see that I am criticising the expression in the articles, and not using it on my own account. Of the value of the letters issued by hospitals, that is open to discussion. In my own constituency about 160 various letters were issued last year, and the subscriptions amounted to about £90. Some forty of these letters were for our Surgical Appliance Committee, about fifty belonged to the dental department, thirteen to skin hospitals, twelve to ophthalmic and six to general hospitals, and the remainder in small quantities between eighteen other institutions. Our subscribers number about 450, so that only one-third of that number needed assistance. I have taken these figures from my own register. Now the Saturday Fund reckons the "benefits" at four to the £1. On this basis my constituency had a balance on the year's working of about £50 for disposal among the medical institutions. My constituents do not subscribe with the idea of getting something in return, but, if unfortunately they require special treatment, it is comforting to think they can get it by applying to the Saturday Fund. Mr. Conrad Thies' assurance that hospital managers are desirous of affording treatment to all serious cases is very pleasant reading.

The Almoner Question.

I will willingly admit that the word I used was injudicious and accept Dr. H. Robinson's better word, "discourteous," therefore I will ask to be allowed to withdraw the former word. The admission that "there may be isolated cases where more sympathy and tact might be shown" I appreciate, and I will allow that almoners, after all, are only human, and at times get irritated by the refusal of the information they are asking for.

Mr. Kemp's contribution chiefly concerns the almoners. He asks me to give names. This would be impossible, as I do not know the names of many almoners, and I think it would be better to avoid the personal equation, but I will quote a few lines from a correspondent's letter: "One of my fellow employees [was refused] hospital treatment [by the lady almoner] because he was earning 30s. per week." The man had a wife and two daughters to keep and a son out of employment. "The department in which he was employed stopped their subscriptions as a protest. . . . This made a great difference to my weekly collection, from which it has never recovered . . . My experience of lady almoners is that they are quite unacquainted with the conditions of life of the average working-class family, and [they] think that 30s. per week for a married man should provide everything—even specialist's fees. I know that some patients under cross-examination by the almoner lie to the best of their ability, knowing that unless they make out a piteous case they will be turned away." In 1908 I reported two cases, but the results were not encouraging, and I lost a lot of subscribers in consequence. This somewhat indicates our difficulties. Whenever I receive an application for a letter I ask the nature of the illness and then recommend the hospital I think best suited to the case. It will happen that the applicant does not agree with my recommendation and chooses for himself. His case is refused and trouble ensues. Subscribers in his department stop payment and it requires an amount of serious talking to get their subscriptions again.

The Distribution Committee's Report.

Mr. Kemp's statement concerning the H.S.F. Distribution Committee's report is rather incomplete, although it is hardly his fault. In the *Hospital Saturday Journal* of December 31, 1912, which he alludes to, on page 6 there is a paragraph under the report of the board meeting of December 5, 1912, in which it is announced "that the Distribution Committee had appointed a special sub-committee to consider and report generally as to the circumstances and conditions attending the reception and treatment of subscribers to this Fund and their dependents at the London voluntary hospitals." Owing to the disfavour with which it was regarded this report was referred to a special committee formed from the six standing committees (two members each), one from each of the local committees, and five members were elected from the delegates then present. This special committee has yet to make its report to the board. At present the delegates have not sanctioned any report on the subject.

The Patients and the Hospitals.

I have now to deal with the excellent and logical letter of Dr. Henry Robinson. I must agree with him that the Insurance Act has contributed to "the disturbance of the old order of affairs." I have referred elsewhere to the almoners. His graceful acknowledgment that "All almoners may not be as tactful and judicious" will, I think, meet the case, and no more need be said. Now, he speaks of the hospital being a charity. The Hospital Saturday Fund is certainly constructed as a provident fund, and that is where we find the divergence of opinion. I do not think that persons who contribute as the working-men subscribers of the Saturday Fund do should be classed with the general body of persons who throng the hospitals for gratuitous treatment. If a working-man subscriber elects to go to a hospital it is not for the treatment of a cough or a boil, but for something of a more serious character. Again, no working man can afford to lose the time required for this purpose, when by going to his local doctor

(*Continued on page* 3, The Institutional Worker.)

### The Saturday Fund and the Hospitals

(continued from p. 576.)

it would cost him much less. I do not overlook Dr. Robinson's reasoning with respect to patients who obtain treatment without the formal intervention of the Saturday Fund, and I must call his attention to the fact that the articles of association of this Fund state "purchase," and I am asking why the word is used. He calls attention to the future problem of hospitals and says, "If Saturday Funds and similar collections break down or cease to exist, the voluntary hospitals may cease to exist too." Now this is exactly my idea, and as a strong upholder of voluntary institutions no one would regret this more than myself. I have devoted many years to the cause of these and other institutions, and I have persuaded many persons to endeavour to make some voluntary provision for the future, not in the nature of a "commercial" contract, but to be obtainable should they require it. If hospitals ceased to exist our medical schools would also cease to exist, and the splendid medical training of the present day would probably disappear and we might get inferior doctors. No; whether we disagree on certain points or not, we must keep the main facts to the front, and I quite think that Dr. Robinson is convinced that I am not throwing mud but am really concerned regarding the existing state of affairs of the present moment, and that I am desirous that a remedy should be found therefor.—I am, Sir, yours very truly, G. A. LEWCOCK.

54 Solon Road, Brixton.

### The Treatment of Tuberculosis.

*To the Editor of* THE HOSPITAL.

